# Effects of Extracorporeal Shock Wave Therapy, Photobiomodulation, Ultrasound, and Electrotherapy Modalities on Rotator Cuff-Related Shoulder Pain: A Systematic Review

**DOI:** 10.3390/jfmk11040383

**Published:** 2026-09-24

**Authors:** Dennis Bühl, Alexandra Unger, Andreas Konrad

**Affiliations:** 1 Human Movement Science, Sport and Health, University of Graz, 8010 Graz, Austria; dennis.buehl@hotmail.de; 2 Department of Sport Science and Health, University of Teacher Education Carinthia, 9020 Klagenfurt am Wörthersee, Austria; alexandra.unger@phk.ac.at

**Keywords:** extracorporeal shock wave therapy, photobiomodulation, laser, ultrasound, electric stimulation therapy, rotator cuff, shoulder impingement syndrome, rotator cuff-related shoulder pain

## Abstract

**Background and Objectives**: The term rotator cuff-related shoulder pain (RCRSP) refers to pain localized in the deltoid region. RCRSP and the associated pain and functional limitations have a significant impact on the quality of life of those affected. This systematic review aimed to investigate the treatment and follow-up effects of extracorporeal shock wave therapy (ESWT), photobiomodulation (PBM), ultrasound (US), and electrotherapy modalities (ET) in RCRSP. **Methods**: A comprehensive literature search was conducted. In addition, the reference lists of the included studies and relevant reviews were screened to identify further eligible studies (snowballing). Randomized controlled trials (RCTs) investigating the treatment and follow-up effects of ESWT, PBM, US, and ET for RCRSP, with control groups receiving sham or alternative physiotherapeutic treatments, were included without restrictions on date or language. Two reviewers independently conducted the literature screening, data extraction, and risk-of-bias assessment for the included studies. The differences in pain between pre- and post-intervention, the between-group differences (intervention vs. control), as well as subgroups (low-level laser therapy [LLLT] and high-power laser therapy [HPLT]) were analyzed. **Results**: A total of 20 RCTs with 1153 patients were included. Quality assessment indicated a low risk of bias in 55% of the included studies, some concerns in 35%, and high risk in 10%. In the nature of a sustainable therapy, therapeutic modalities were mostly not applied in isolation but rather in combination with conventional physiotherapeutic interventions. One study showed an end-of-treatment pain reduction for ESWT, and one study showed no follow-up effect on pain. PBM showed a significant but small end-of-treatment effect and an insignificant follow-up effect. The subgroup analysis suggested that only HPLT demonstrated a clear end-of-treatment effect. Due to the limited number of studies, no subgroup analysis could be performed for follow-up outcomes. US showed beneficial small effects both at the end of treatment and at follow-up, while ET demonstrated an end-of-treatment effect. Further studies are needed for the follow-up effect. **Conclusions**: HPLT, US, and ET showed consistent additive treatment effects, while PBM and US showed follow-up effects. In general, especially for PBM, ESWT, and ET, more studies are needed to gain a broader insight into this topic.

## 1. Introduction

Rotator cuff-related shoulder pain (RCRSP) is a clinical term for symptoms that relate to the deltoid region [1]. It is an umbrella term for various conditions affecting the rotator cuff and the subacromial space, including tendinopathies, partial tears, and subacromial impingement syndrome [2]. However, calcific rotator cuff tendinopathy should be regarded as a distinct clinical condition from RCRSP because it involves calcium deposition, with a unique pathophysiology, clinical course, and management [3]. Patients affected with RCRSP typically experience pain during arm use, particularly with elevation or abduction, in the absence of neurological involvement [1]. The pain in RCRSP has a multifactorial etiology [4,5,6,7], with certain components arising from local biological mechanisms. Various biomolecules are directly linked to pain perception. Thus, substance P, various interleukins, vascular endothelial growth factor, and tumor necrosis factor in the shoulder contribute to the experience of pain [5,6]. The diagnosis RCRSP recognizes that the exact origin of the pain is currently not fully understood [1]. RCRSP is primarily diagnosed clinically (e.g., based on pain provocation tests, assessments of mobility and strength), as the diagnosis relies on the reproduction of symptoms in combination with functional deficits rather than on imaging findings alone [8]. To some extent, it represents a diagnosis of exclusion [1]. Imaging techniques, such as magnetic resonance imaging (MRI), may be used to support the clinical assessment and to rule out full-thickness rotator cuff tears when indicated [8].

Risk factors for rotator cuff tendinopathy, and consequently for RCRSP, include demographic factors such as age over 50 and male sex, metabolic and lifestyle conditions such as diabetes, smoking, and obesity, and relevant medical conditions, including previous shoulder injuries, musculoskeletal imbalances, and autoimmune disorders [9]. Occupational risk factors include heavy manual work, repetitive work, high frequency of work, high force exposure of work, and vibration work [9]. The prevalence in the general population worldwide is around 2 to 7% [10]. Individuals with general shoulder pain not only report more severe pain and functional limitations but also exhibit a higher prevalence of psychological distress. Among people with general shoulder pain, 22.3% suffer from depression, 19.2% suffer from anxiety, and 81.5% report sleep disorders, while the prevalence of these complaints is significantly lower in people without shoulder pain (depression 8.4%, anxiety 10%, sleep disorders 25%) [11]. This underscores the clinical relevance of pain reduction in RCRSP, as mitigating pain symptoms is essential to reducing the associated psychological burden and improving the overall quality of life for affected patients.

There are a variety of non-surgical treatment options for RCRSP, such as medications, resistance training, motor control exercise, stretching, education, kinesiotaping, and dry needling [12,13,14]. For example, strength training is capable of reducing pain, while scapular stabilization exercises have been shown to improve pain symptoms in RCRSP too [13]. Additionally, medications such as oral non-steroidal anti-inflammatory drugs can provide pain relief [14]. Non-surgical therapies also include device-assisted modalities, such as extracorporeal shock wave therapy (ESWT), photobiomodulation (PBM), ultrasound (US), and electrotherapy modalities (ET) [15,16,17,18]. Crucially, the clinical rationale for utilizing these device-assisted modalities extends beyond symptomatic pain relief, as they are proposed to directly target the underlying structural pathology. It must be noted that RCRSP can also involve structural changes in the rotator cuff tendons [19,20]. For example, tendon degeneration can occur. This includes, among other things, loss of fine collagen fiber structure and parallel arrangement, and increased cellularity and vascularity [20]. Except for ET, the aforementioned device-assisted modalities have the potential to modify the structure of pathological tendons [21,22,23,24,25]. This makes them high-value therapies because of their potential to intervene in the fundamental processes of RCRSP.

The ESWT device produces shock waves [26]. A shock wave is a pressure wave with a frequency spectrum in the range of 16 Hz to 20 MHz [27]. These shock waves can affect the microstructure of the tendon, potentially altering cell shape and increasing collagen production [25].

PBM includes different laser types [28]. Low-level lasers have a power output of less than 0.5 W, whereas high-power lasers exceed 0.5 W. The laser light penetrates tissues and, in the process, is reflected, scattered, and absorbed. Depending on the power and wavelength, the laser light penetrates the tissue to different depths [29]. Low-level laser irradiation is capable of increasing collagen synthesis in tenocytes, stimulating mitochondrial activity, and increasing the viability of these cells [23,24,26,30].

US is a procedure in which ultrasound waves are generated. It has thermal and nonthermal effects on the tissue. It may play a role in the proliferation of fibroblasts, collagen production, and remodeling of the damaged tissue [31].

ET is an umbrella term that encompasses various modalities, such as transcutaneous electrical nerve stimulation (TENS), interferential current, etc. [18]. ET can work deep within the tissue. Possible mechanisms of action include increased circulation and the blocking of nerve conduction [30].

The existing evidence on some of the individual modalities has shown heterogeneous results regarding their efficacy on pain [8,16,32,33,34,35,36,37,38,39,40,41]. Although systematic reviews and meta-analyses on individual procedures such as ESWT or laser therapy already exist [15,42,43,44], a comprehensive overview synthesizing and juxtaposing the evidence across different device-assisted modalities has yet to be conducted. Furthermore, in physiotherapeutic practice, these interventions are often not used in isolation but in combination with other therapies, which further complicates the interpretation of their specific effects.

The aim of this study was therefore to systematically evaluate the end-of-treatment and follow-up effects of ESWT, PBM, US, and ET on pain in patients with RCRSP. Randomized controlled trials comparing these modalities with placebo/sham or non-surgical interventions were included, as they provide the most robust available evidence for clinical evaluation. To minimize confounding, co-interventions (e.g., physiotherapy) had to be applied consistently across experimental and control groups, thereby allowing the assessment of the specific effects attributable to each modality.

## 2. Methods

### 2.1. Protocol and Registration

This systematic review was conducted in accordance with the PRISMA (Preferred Reporting Items for Systematic Reviews and Meta-Analyses) guidelines [45] and was registered in the PROSPERO database (https://www.crd.york.ac.uk/PROSPERO/view/CRD420251074764; accessed on 27 June 2025) under ID CRD420251074764.

### 2.2. Eligibility Criteria

This study included RCTs that examined the effectiveness of ESWT, PBM, US, and ET in managing RCRSP. (1) Patients with symptoms related to RCRSP, irrespective of duration or specific conditions encompassed by this umbrella term (i.e., non-calcific rotator cuff tendinopathy, rotator cuff tendinosis, rotator cuff tendinitis, supraspinatus tendinitis, rotator cuff injury, rotator cuff disease, supraspinatus tendon syndrome, subacromial syndrome, periarticular shoulder pain, subacromial impingement, subacromial pain syndrome, subacromial bursitis, subacromial bursopathy, or partial-thickness rotator cuff tear). Only non-calcific rotator cuff tendinopathies were included. (2) The experimental group underwent ESWT, PBM, US, or ET, while the control group received a placebo/sham and/or non-surgical intervention (including different non-surgical interventions and different application techniques or doses of the same non-surgical modality). In addition, the experimental group may have also received physiotherapeutic interventions. In this case, the control group must have also received physiotherapeutic interventions to estimate the net effect of ESWT, PBM, US, or ET. US and ET could also have been administered through phonophoresis or iontophoresis. (3) The primary outcome was pain, assessed using the visual analog scale (VAS), Shoulder Pain and Disability Index (SPADI) (pain), Constant-Murley score (CMS) (pain), numerical rating scale (NRS), Short Form 36 (SF36) (bodily pain), and Nottingham Health Profile (NHP) (pain). If data were not provided in the corresponding paper, the authors were contacted. (4) There were no restrictions regarding language or the publication date. A detailed description of the outcome variables is provided in Table 1.

### 2.3. Exclusion Criteria

Exclusion criteria were as follows: (1) Previous surgery on the examined side. The same intervention was previously performed. Intervention within three months on the examined side. Full-thickness rotator cuff tear/s, calcification of the rotator cuff, involvement of the long head biceps tendon (except in cases where the treatment did not specifically address the biceps muscle), osteoarthritis, fractures, shoulder instability, labral tear, adhesive capsulitis, systemic inflammation, or neurological diseases (also the absence of referred pain) and rheumatological conditions. The subjects’ use of anti-inflammatory or antipyretic drugs was not controlled. (2) Surgical interventions were combined or studied exclusively. (3) Non-human studies. (4) Absence of pre-and post-comparisons, if only other parameters as outcome variables were measured. (5) Letters, reviews, case reports, conference abstracts, and comments, i.e., non-RCTs.

If the relevant outcome measures were missing, the authors were contacted. If no outcome change could be calculated from the available data (due to the lack of necessary data), the corresponding study was excluded.

### 2.4. Retrieval Strategy

Searches across all databases were performed on 10 July 2025, capturing all available literature from inception up to that date. The following databases were searched: PubMed, Scopus, and Web of Science (WoS). No search limits were applied to any database. The search strategy involved a combination of subject terms and free-text keywords, utilizing Boolean operators (AND/OR). The specific search approach varied slightly between databases. The following search code was used: (“subacromial pain syndrome” OR “subacromial impingement syndrome” OR “rotator cuff” OR “supraspinatus” OR “infraspinatus” OR “subscapularis” OR “teres minor” OR “shoulder pain” OR “subacromial pain” OR “shoulder tend*” OR “shoulder burs*” OR “subacromial burs*”) AND (“laser” OR “extracorporeal shockwave therapy” OR “shock” OR “ultraso*” OR “electr*” OR “transcutaneous electrical nerve stimulation” OR “TENS” OR “photobiomodulation”) AND (“controlled” OR “trial” OR “randomized” OR “randomised” OR “effect*” OR “RCT” OR “randomly” OR “clinical trial”) NOT (“children” OR “kids” OR “animal*” OR “corticosteroid” OR “steroid” OR “inject*” OR “platelet rich plasma” OR “labral” OR “instability” OR “frozen shoulder” OR “capsulitis” OR “arthritis” OR “osteoarthritis” OR “ultrasound-guided percutaneous irrigation” OR “surgery” OR “surgical” OR “operative” OR “review” OR “meta analysis” OR “stroke” OR “epicon*” OR “patellar” OR “achilles” OR “neur*” OR “cervical” OR “spin*” OR “neck”).

### 2.5. Study Selection

All studies were imported into an Excel (Microsoft Corporation, Redmond, WA, USA) spreadsheet, where duplicates were identified and removed using a deduplication function. Two reviewers (D.B., A.U.) then examined each study’s title and abstract to evaluate its relevance according to the established inclusion and exclusion criteria. In the case of conflicts, a third reviewer was consulted (A.K.). The full texts were then read. Studies that did not meet the inclusion criteria were discussed and excluded if necessary. After this step, a snowballing of the included studies was carried out.

### 2.6. Data Collection Process

Two researchers (D.B., A.U.) extracted the data. The following data were extracted: (1) general information: author, year, country, number of participants/shoulders, women-to-men ratio, mean age, and mean duration of symptoms; (2) specific therapeutic parameters: interventions, localization/area, dose, duration, frequency, and number; (3) outcome measures: outcome measures (pain and time), assessment tools, and side effects; (4) outcomes were classified as end-of-treatment effects (immediately after the end of an intervention) and follow-up effects (≥ two weeks).

### 2.7. Data Analysis

The primary outcome was pain, which could be measured using various assessments. When studies reported outcomes as medians alongside interquartile ranges, means were estimated using the validated methods of Hozo et al. [46] or Wan et al. [47], depending on the reported summary statistics. Standard deviations were available for all included studies. If a study reported multiple distinct pain measures (i.e., VAS/NRS, SPADI–pain, CMS–pain, SF36, or NHP–pain), each instrument was extracted and analyzed as an independent variable. No mathematical aggregation or pooling of multiple pain scores within the same study was performed. If the same measurement instrument was used under varying conditions (i.e., night, activity, and rest pain), an arithmetic mean score was calculated across these conditions to generate a single composite value per study. This approach was chosen to prevent unit-of-analysis errors from dependent data within the same participant cohort. To assess end-of-treatment effects, we calculated the change scores between the pre-measurement and the measurement taken immediately upon completion of the intervention. For follow-up effects, the difference was calculated between the pre-measurement and the final follow-up measurement, which occurred at least two weeks after the intervention ended. To quantify the net effect of each intervention, we calculated the difference in change scores between the intervention and control groups. In case fewer than three studies were available, a qualitative description of the study was provided. If more than two studies were available for a given outcome and time point, weighted mean changes were calculated. The weighted value was determined by dividing the number of participants in a given study by the total number of participants. The weighting factor for each study was determined by dividing its sample size by the total number of participants across the included studies. This weight was then multiplied by the mean pre-to-post change score of the respective study. This procedure was performed separately for the intervention and control groups. Subsequently, the weighted change score of the control group was subtracted from that of the intervention group to obtain the final weighted mean changes. In addition, the sum of all weighted mean changes was calculated.

In addition, the corresponding 95% confidence intervals (CIs) were calculated with a student’s t-distribution as follows:

(1) $$95\%\;\mathrm{CI}={\bar{X}}_{\mathrm{weighted}}\pm t_{0.975,\;k-1}\cdot \frac{s}{\sqrt{k}}$$

where ${\bar{X}}_{\mathrm{weighted}}$ is the overall weighted mean, s is the sample standard deviation of the weighted study-level changes, k is the number of included studies for the respective outcome, and $t_{1-\alpha /2,\;k-1}$ is the critical t-value at α=0.05 with k−1 degrees of freedom.

If a study investigated more than one similar device-assisted modality, the mean was calculated and used for further statistical analyses. Subgroup analyses were conducted whenever more than two studies were available per subgroup. In these cases, the weighted mean changes were re-estimated for each subgroup. If data were missing, the authors were contacted and asked to provide the missing data.

While a meta-analysis could have offered more quantitative insights, the substantial heterogeneity in the outcome measures across the included studies precluded a meaningful data synthesis. Although meta-analyses can be theoretically performed using ordinal- and scale-based outcomes, the variety of scales, indices, and questionnaires used across the studies posed significant challenges for meaningful comparison or analysis.

### 2.8. Study Risk-of-Bias Assessment

Two researchers (D.B., A.U.) independently evaluated the risk of bias in the included studies using the Cochrane risk of bias 2 (RoB 2) [48]. Disagreements regarding risk-of-bias assessments were resolved through discussion or, if necessary, by consulting a third author (AK). Risk of bias was evaluated across the five standard RoB 2 domains addressing trial design, conduct, and reporting: bias arising from the randomization process, bias due to deviations from intended interventions, bias due to missing outcome data, bias in outcome measurements, and bias in the selection of the reported result.

## 3. Results

### 3.1. Study Selection

A total of three databases were searched, yielding 2716 initial records. Following the removal of duplicates using a deduplication function, 1574 studies remained. Title and abstract screening by two independent reviewers further reduced the number to 50. After full-text assessment, 31 studies were excluded. A total of four reports could not be retrieved for full-text evaluation. Despite contacting the primary authors via email, no responses or full-text copies were obtained, and these records were consequently excluded. Snowballing revealed five additional studies. Finally, 20 studies were included (Figure 1).

### 3.2. Study Characteristics

Details of the included studies are summarized in Table 2. A total of 20 studies were included: two studies investigated the ESWT only; eight studies investigated the PBM only; two studies investigated the PBM and US simultaneously; two studies investigated the US only; two studies investigated the US and ET simultaneously; four studies investigated the ET only. In total, 1153 eligible patients were included across the studies, with 64 receiving ESWT, 255 PBM, 128 US, and 137 ET (584 in total received interventions and 569 were in the control groups). Across all included studies, only one shoulder was evaluated per participant (one participant=one shoulder). Consequently, all reported outcomes reflect single-shoulder interventions.

Eleven studies examined the end-of-treatment effects and three studies examined the follow-up effects; six studies examined both effects. There was heterogeneity in assessment methods. The VAS was measured differently: as activity/movement, nocturnal, rest, or without specification. The NRS was measured as daily life activities or rest. The SPADI, NHP, CMS, and SF36 were used in some studies for assessment, with the pain subscale being utilized. The most commonly used assessments were the VAS (16 times) and the SPADI (eight times). The NHP, CMS, SF36, and NRS were used one, three, one, and one time, respectively. Both the intervention and control groups primarily received conservative treatments [8,16,18,32,33,34,35,36,37,38,39,40,41,50,51,52,53,54,55], with sham interventions being added in eight studies [8,18,34,36,39,41,51,52]. These conservative protocols frequently involved combinations of various modalities, such as exercise therapy and stretching. Notably, one ESWT control group received a sham intervention as the sole comparator [56]. Further details on the conditions are provided in Table 2.

**Table 2:** Study characteristics of the included ESWT (*n* = 2), PBM (*n* = 9), US (n = 6), and ET (*n* = 6) studies.

Study	Number of Participants (n)/Shoulders (n) (Women/Men) {Mean Age (Years)} {Mean Duration of Symptoms (Months)}	IG	CG	Localization/Area	Dose	Duration (min)	Frequency, Numbers	Side Effects
**ESWT**								
Pavlovskiy et al. [50], Russia	60/60 {50.97 ± 5.2} {NR}	ESWT + OT (with ET)	OT (with ET)	Working head diameter: 15 to 40 mm Deep penetration applicator (working head diameter 15 mm) over areas of maximum tenderness, followed by scanning D-actor 20 mm applicators. Wider coverage of affected areas up to healthy tissues: V-actor II 40 and 20 mm applicators	Radial shock waves, force of impact 2.5 to 5.0 bar, frequency 8 to 10 Hz, deep penetration applicator, scanning (1.6 to 2.8 bar, 12 to 17 Hz), movement speed 2–4 cm/s and wider coverage (1.8 to 2.0 bar, 25 to 31 Hz), 6000 impulses per procedure The procedure duration was 12–15 min, with a total exposure of 6000 impulses per procedure The interval between procedures was 7 days, with 3 procedures per course	12 to 15	Three sessions, interval between procedures 7d	No
Speed et al. [56], England	74(43W/31M) {IG = 50.7, CG = 54.2) {NR}	ESWT	ESWT sham	Site of maximal tenderness	1500 pulses; 0.12 mJ/mm^2^	NR	1/m, three sessions	Could not tolerate the therapy (one) or for unknown reasons (three)
**Laser**								
Sen et al. [32], Turkey	36/36(28W/8M) {IG = 52.2 ± 8.3, CG = 54.7 ± 10.7} {IG = 9.1 ± 8.4, CG = 8.3 ± 7.1}	Laser + OT (LLLT)	OT	Five points over the shoulder, over the greater and lesser tubercles, the bicipital groove, and the anterior and posterior aspects of the capsule	Wavelength 850 nm; power 100 mV *; continuous wave; dose 3 J/cm^2^	One at each point, 5 total	5/w, 1/d, 15 sessions	NR
Beyrami et al. [33], Iran	32(15W/17M) {IG = 46 ± 8.53, CG = 39 ± 8.68} {NR}	Laser + OT (with US + ET) (HPLT)	OT (with US + ET)	Tender points around the shoulder joint	Average power 4 W; wavelength 808 nm; energy density 20 J/cm^2^; spacer 2.5 cm	0,33 per point, 3,33 in total	12 sessions	NR
Alfredo et al. [16], Brazil	84/84 {IG = 51.9 ± 8.7, CG = 54.2 ± 7.1} {NR}	Laser + OT (LLLT)	OT	Over supraspinatus muscle tendon, subacromial bursa, along the bicipital groove	3 J per point, total dose 27 J per treatment; wavelength 904 nm; frequency 700 Hz; average power 60 mW; peak pulse power 20 W; area 0.5 cm^2^	50 s irradiation per point	3/w, 8w	NR
Elsodany et al. [34], Arabia	60 {50.2 ± 3.6} {NR}	Laser + OT (HPLT)	Laser sham + OT	Over rotator cuff muscles, upper fibers of the trapezius, deltoid, and pectoralis major muscles	Treatment consisted of initial, intermediate, and final phases; fast scanning in the initial phase and slow scanning in the final phase, total energy of 1000 J each phase, intermediate phase, and laser probe fixed to predetermined trigger and tender points at 90° perpendicular to skin. Mean energy 50 J; total energy during one session, 2050 J over three phases of treatment, calibrated for constant output	15	3/w, 4w, 12 sessions	NR
Dogan et al. [8], Turkey	52/52(33W/19M) {IG = 53.7 ± 12.6, CG = 53.45 ± 9.64} {IG = 11.66 ± 18.04, CG = 15.27 ± 25.13}	Laser + OT (LLLT)	Laser sham + OT	0.07 cm^2^ spot area, subacromial region of the shoulder	Wavelength 850 nm; power output 100 mV *; continuous wave; dosage 5 J/cm^2^ (totaling 15 to 20 J); maximum 5 to 6 painful points	5 to 6, 1 each point	5/w, 14 sessions	No
Calis et al. [35], Turkey	31(21W/10M) {IG = 46.2 ± 12.14, CG = 50.34 ± 13.69} {IG = 3, CG = 3}	Laser+ OT (LLLT)	OT	Shoulder	904 nm wavelength; 6 mW average power; 1 J/cm^2^ dosage; 16 Hz frequency	5	Daily, 15 sessions	NR
Yeldan et al. [36], Turkey	60/60(47W/13M) {IG = 55.32 ± 8.73, CG = 55.00 ± 8.75} {IG = 6.50 ± 4.52, CG = 6.42 ± 4.79}	Laser + OT (LLLT)	Laser sham + OT	Superior and anterior periarticular parts; glenohumeral joint covering area approx. 15 cm^2^; maximum five tender points, subacromial and anterior shoulder regions	Each session consisted of three pulses (3 J); frequency 2000 Hz; wavelength 904 nm; frequency range of 5 to 7000 Hz; maximum peak power of 27, 50, or 2764 W	90 s at each location, approx. 8	5/w, 3w 15 sessions	No
Pekyavas and Baltaci [37], Turkey	35 {IG = 51.1 ± 14.3, CG = 45.4 ± 15.5} {NR}	Laser + OT (HPLT)	OT	Bright spot diameter 5 mm; three phases of treatment: starting, trigger, and closing each session; initial phase zones: muscular contracture, particularly upper trapezius, supraspinatus, and deltoid muscles; anteriorly, pectoralis minor muscle during abduction shoulder position, scanning transverse and longitudinal directions with arm positioned in internal rotation and extension. Trigger points including four phases for each point; final phase, slow manual scanning (100 cm^2^/60 s) of same areas treated in initial phase	Pulsating waveform, high power peaks (3 kW), pulsed source (wavelength 1064 nm), initial phase: fast manual scanning (100 cm^2^/30 s); this phase has a total energy dose of 1000 J/cm^2^; intermediate phase on trigger points, including four phases for each point until pain reduction of 70 to 80% is achieved in 6 s. This phase has a mean energy dose of 50 J; final phase: slow manual scanning (100 cm^2^/60 s) until a total energy dose of 1000 J, 3 steps predicted in starting/initial and final phases, fluencies 510, 610, and 710 mJ/cm^2^, with a total dose energy of ~ 2050 J	~30	3/w, 15 sessions	NR
Bal et al. [38], Turkey	40/40(28W/12M) {IG = 51.7 ± 14.1, CG = 53.1± 8.4} {NR}	Laser + OT (LLLT)	OT	Over tuberculum majus and minus, anterior and posterior faces of the capsule, subacromial regions	Wavelength 904 nm; 5500 Hz frequency; 27 W maximum power output per pulse; 13.2 mW average power; 0.8 cm^2^ spot size; 1.6 J total energy delivered per point each session at a power density of 16.5 mW/cm^2^; cumulative energy per point all sessions 16 J	10, each point 2	5/w, 2w	No
Öztürk et al. [39], Turkey	60/60(45W/15M) {IG = 51.3 ± 8.7, CG = 54.5 ± 7.2} {IG = 5.5, CG = 8}	Laser + OT (with ET) (LLLT)	Laser sham + OT	Three points (subacromial space, supraspinatus tendon insertion, and glenohumeral joint), 0.07 cm^2^ spot area	Dose: 4 J (total 12 J), wavelength 850 nm, power output 100 mW, continuous wave	40 s (total of 120 s) to each of 3 points	5/w, 15 sessions	No
**US**								
Sen et al. [32], Turkey	35/35(23W/12M) {IG = 48.5 ± 11.9, CG = 54.7 ± 10.7} {IG = 6.2 ± 6.1, CG = 8.3 ± 7.1}	US + OT	OT	NR	Transducer head size of 5 cm^2^; frequency 1 MHz; power 1.0 W/cm^2^; pulsed	5	NR	NR
Kelle et al. [51], Turkey	66/66(49W/17M) {IG = 44.8 ± 6.9, CG1 = 50.7 ± 8.8, CG2 = 49.1 ± 9.2} {NR}	US + OT continuous mode and US + OT pulsed mode (phonophoresis)	US sham + OT	Effective radiation area of 5 cm^2^ and effective radiation area of 5 cm^2^	Ibuprofen cream 5 g (1 g containing 50 mg active ingredient); frequency 1 MHz, intensity 1 W/cm^2^; US head perpendicular contact and ibuprofen cream 5 g (1 g containing 50 mg active ingredient); frequency 1 MHz, intensity 1 W/cm^2^; US head perpendicular contact	5 and 5	5/w, 3w, 15 sessions each intervention Grp	No
Ucurum et al. [40], Turkey	39(36W/3M) {IG = 48.55 ± 7.89, CG = 47.89 ± 7.12} {NR}	US +OT	OT	Around affected shoulder	1 MHz; intensity 1.5 W/cm^2^	5	3/w, 3w	NR
García et al. [52], Spain	56(34W/22M) {IG = 61.9 ± 13.0, CG = 56.9 ± 12.3} {NR}	US + ET sham + OT	US sham + ET sham + OT	Effective radiation area in study: 5 cm^2^; treated area four times the effective radiation area; beam nonuniformity radio less than 5; insertion of supraspinatus; infraspinatus insertion	1 MHz; continuous mode (100%); 0.7 W/cm^2^; sodium diclofenac gel as topical medium	5	5/w, 3w, 15 sessions	No
Calis et al. [35], Turkey	37(25W/12M) {IG = 50.42 ± 12.41, CG = 50.34 ± 13.69} {IG = 3, CG = 3}	US + OT	OT	Probe: 20 mm diameter; shoulder	Power 1.5 W/cm^2^; frequency 3 MHz; continuously circular mode	5	Daily, 15 sessions	NR
Kılıç et al. [53], Turkey	40(20W/20M) {IG = 52.50 ± 7.00, CG = 51.15 ± 6.87} {IG = 7.03 ± 4.25, CG = 6.05 ± 3.60}	US + OT	OT	Surface head 5 cm^2^; rotator cuff	Frequency 1 MHz; intensity 1.5 W/cm^2^; continuously, circular motion	8	3/w, 4 w, 12 sessions	NR
**ET**								
Tugay and Kul [18], Turkey	52/52(43W/9M) {IG = 51.8 ± 8.8, CG1 = 51.8 ± 12.1, CG2 = 49.1 ± 12.3} {IG = 9.9 ± 10.6, CG1 = 9 ± 8.6, CG2 = 5.9 ± 8.2}	ET + OT and ET +OT (TENS and IFC)	sham ET + OT	Four electrodes across the glenohumeral joint—glenohumeral joint in the center and painful shoulder region	Amplitude 80 to 150 Hz and pulse length 20 to 60 μs; stimulation frequency 95 Hz	20 and 30	5/w, 15 sessions each intervention Grp	No
Koumantakis et al. [54], Greek	16 {49} {NR}	ET + OT	OT	Three parts deltoid muscle	Intensity 30% capacitive current head; intensity 40% deep capacitive current function head; intensity 40% resistive form current head; frequencies between 300 KHz and 1 MHz	Five capacitive current, five deep capacitive current, five resistive form current, total of 15	8w, 12 sessions, 2/w first 4 w, 1/w next 4 w	NR
Ucurum et al. [40], Turkey	59(46W/13M) {IG = 47.70 ± 6.51, CG1 = 48.50 ± 8.34, CG2 = 47.89 ± 7.12} {NR}	ET + OT and ET +OT (IFC and TENS)	OT	Around affected shoulder and around affected shoulder	50 to 120 Hz frequency and conventional mode	20 and 20	3/w, 3w	NR
García et al. [52], Spain	51(32W/19M) {IG = 57.7 ± 17.3, CG = 56.9 ± 12.3} {NR}	ET + US sham + OT	US sham + ET sham + OT	Placement alternated: one day of active electrode placed on the insertion site of the supraspinatus tendon, with the non-active electrode placed on infraspinatus insertion; the next day electrodes were placed on opposite insertion sites	5 × 5 electrodes, intensity 0.1 to 0.2 mA/cm^2^; sodium diclofenac (75 mg vials of diclofenac), administered under active electrode (cathode)	10	5/w, 3w, 15 sessions	No
Rani et al. [55], India	70/75(38W/32M) {IG = 47.48 ± 13.75, CG = 47.28 ± 9.91} {NR}	ET + OT	OT	NR	Conventional mode; high frequency 100 Hz; pulse duration 120 μs; low intensity 30 to 40 mA	20	1/d, five consecutive d, 5 sessions	No
Nazligul et al. [41], Turkey	60/60(34W/26M) {IG = 51.13 ± 8.80, CG = 48.90 ± 9.40} {IG = 5.12 ± 5.78, CG = 5.36 ± 6.79}	ET + OT	sham ET + OT	Electrodes 90 × 50 mm	Bipolar, medium frequency 4000 Hz and 4100 Hz to produce amplitude-modulated frequency at 100 Hz; physiotherapist increased current amplitude until participant felt strong but comfortable tingling; every 5 min, physiotherapist asked participant whether “strong but comfortable tingling” remained; in case of sensory habituation, current amplitude was increased until participant reached previous sensation	20	5/w, 10 sessions	No

D = day/s, w = week/s, m = month/s, OT = other treatments, Grp = group, IG = intervention group, CG = control group, TENS = transcutaneous electrical nerve stimulation, LLLT = low-level laser therapy, HPLT = high-power laser therapy, NR = not reported, * original report states mV, though mW (milliwatts) is the likely intended unit for power; original report states beam nonuniformity radio, though beam nonuniformity ratio is likely intended.

### 3.3. Results of the Quality Assessment

The 20 included RCTs underwent risk-of-bias assessment. Two studies showed a high risk of bias. Overall, the studies showed some concerns regarding bias (Figure 2 and Figure 3).

### 3.4. Results of the Intervention Types

#### 3.4.1. ESWT

Only one eligible study assessed the end-of-treatment effect, and one study assessed the follow-up effect. Pavlovskiy et al. [50] evaluated the end-of-treatment pain-relieving effects of ESWT compared to other treatments. ESWT demonstrated superior pain outcomes, achieving a statistically significant 0.5 cm reduction in VAS compared to other interventions. The study included 30 participants in the ESWT group and 30 in the control group. Speed et al. [56] investigated the follow-up pain effects of ESWT (*n* = 34) against a control group (*n* = 40) that received a sham ESWT intervention. They reported an insignificant difference of −0.08 cm in favor of the control group.

#### 3.4.2. PBM

Considering PBM, nine studies assessed end-of-treatment effects [8,16,33,34,35,36,37,38,39], while four studies reported follow-up effects [16,32,34]. In all the included studies, PBM was not applied as a standalone treatment but was combined with other physiotherapeutic interventions. Compared with the control groups, PBM demonstrated greater end-of-treatment pain relief, as measured by VAS, across nine studies (weighted mean change = 0.43 cm, 95% CI: 0.31 to 0.55) [8,16,33,34,35,36,37,38,39]. Across five studies, end-of-treatment SPADI–pain scores improved in favor of the intervention compared with the control (weighted mean change = 3.99%, 95% CI: 0.08 to 7.9) [8,16,33,37,38]. One study assessed end-of-treatment pain using the CMS–pain subscale and found an insignificant 0.44-point difference, favoring the control group over the intervention [36]. A subgroup analysis showed that the difference for end-of-treatment pain VAS (weighted mean change = 2.18 cm, 95% CI: 1.03 to 3.33, three studies) was in favor of HPLT [33,34,37], and for LLLT (weighted mean change = −0.23 cm, 95% CI: −0.42 to −0.04, six studies), it was in favor of the control group [8,16,35,36,38,39]. Based on four studies assessing the follow-up effects on VAS, a pain reduction was reported (weighted mean change = 1.13 cm, 95% CI: −0.29 to 2.55) compared to the control group [16,32,34]. Two studies reported an insignificant end-of-treatment decrease in pain on the SPADI (pain subscale) [16,32]. Sen et al. [32] showed a difference of 7.6 points and Alfredo et al. [16] reported a difference of 24.3 points compared to the control group.

#### 3.4.3. US

For US, end-of-treatment effects were assessed in four studies [35,40,51,53], while follow-up effects were examined in five studies [32,40,51,52,53]. Across all the included studies, US was never applied as a standalone treatment, but it was always part of a combination with other physiotherapeutic methods. In contrast with the control groups, US demonstrated greater end-of-treatment pain relief, as measured by the VAS, across four studies (weighted mean change = 0.72 cm, 95% CI: 0.46 to 0.98) [35,40,51,53]. One study assessed the end-of-treatment pain relief with the Nottingham Health Profile (NHP) questionnaire (pain subscale) and showed a significant 9.1-point difference, favoring US over the control group [51]. Compared to the control group, a follow-up pain reduction was observed in four studies on the VAS (weighted mean change = 0.88 cm, 95% CI: 0.67 to 1.09) [32,40,51,53]. One study showed a significant follow-up pain reduction on the NHP (pain subscale) with a difference of 4 points [51]. One study used the CMS (pain subscale) and SF36 (bodily pain subscale) to assess follow-up pain relief and found an insignificant 3.1-point and significant −13.5-point difference, favoring US [52]. One study showed an insignificant follow-up pain reduction on the SPADI (pain subscale), with a difference of 6.4%.

#### 3.4.4. ET

The end-of-treatment effects of ET were assessed in five studies [18,40,41,54,55], whereas its follow-up effects were examined in three [40,41,52]. ET was always compared with other interventions, except in Tugay and Kul [18], who compared ET only to a sham ET. Based on three studies, ET showed greater end-of-treatment pain relief compared to the control groups on the VAS (weighted mean change = 1.55 cm, 95% CI: 0.41 to 2.69). Two studies used the SPADI (pain subscale) for end-of-treatment pain assessment [54,55]. Koumantakis et al. [54] found an insignificant difference of −3.7% favoring the control group, and Rani et al. [55] showed a significant difference of 35.33% favoring ET [55]. For the follow-up pain assessment, two studies used the VAS [40,41]. Ucurum et al. [40] reported a difference of 0.02 cm, and Nazligul et al. [41] reported a difference of 2.45 cm in favor of ET. The findings in both cases were not statistically significant. Two studies used the CMS (pain subscale) to assess the follow-up pain [41,52]. García et al. [52] reported a difference of 1 point favoring ET, and Nazligul et al. [41] reported a difference of −0.5 points favoring the control group, but the results did not reach statistical significance. García et al. [52] assessed the follow-up pain using the SF36 (bodily pain subscale) and found a difference of −7.6 favoring ET, but the result was not statistically significant (see Table 3).

Most studies did not report any adverse effects or stated that there were none. Regarding ESWT, one study reported that a subject could not tolerate the therapy [56], but they did not provide any detailed information about this. For PBM studies, half reported nothing [16,32,33,34,35,37], and the other half reported that there were no adverse effects [8,36,38,39]. The majority of US studies reported nothing [32,35,40,53], and the others reported no adverse effects [51,52]. Regarding ET studies, most reported no adverse effects [18,41,52,55], while others reported nothing [40,54]. Despite the lack of reporting, it appears that ESWT was the only intervention associated with side effects. The reported side effects could be described as minor.

## 4. Discussion

RCRSP is a heterogeneous clinical syndrome that typically manifests as pain in the deltoid muscle area [1] and can involve various underlying pathological structures [2]. A wide range of therapeutic approaches have been proposed [12]. This systematic review focused on four commonly used interventions for RCRSP [12]: ESWT, PBM, US, and ET. The primary endpoint was pain intensity, which was measured in the included studies using validated instruments. Pain changes relative to baseline were contrasted between intervention and control groups to evaluate end-of-treatment effects, as well as follow-up effects. The results are intended to inform users of the device-assisted modalities about their benefits in pain management and intervention parameters. Furthermore, research gaps should be addressed. Most device-assisted modalities demonstrated positive effects. It should be noted that the interventions were mostly administered in conjunction with other treatments; therefore, the observed results primarily reflect additive effects. However, further studies are needed to evaluate follow-up outcomes for ESWT (both end-of-treatment and follow-up effects), the HPLT subgroup, and ET (Figure 1).

### 4.1. ESWT

The evidence for ESWT in non-calcific RCRSP is limited overall. Numerous studies [57,58,59,60,61,62,63,64] have investigated the topic, but many did not meet our inclusion criteria, particularly due to previous treatments within the last three months. Consequently, the evaluation of ESWT in this systematic review was based on only a very small number of suitable studies, which limits the significance of the results. Pavlovskiy et al. [50] reported a significant end-of-treatment effect, with a VAS difference of 0.5 cm in favor of ESWT. However, due to the number of participants in the intervention group (*n* = 30), it is unclear whether this effect is robust or clinically relevant. In addition, they reported the use of a radial ESWT with a deep penetration applicator. This raises the question of whether the deep structures were adequately stimulated with the radial ESWT [15].

Speed et al. [56] observed a minimal difference of −0.08 cm in favor of the control group during follow-up, which was not statistically significant. In this study, the type of ESWT (radial or focused) was not specified. However, the choice of the correct applicator is crucial, as the penetration depth determines its ability to target the underlying pathophysiology [15]. In addition, only one intervention per month was performed, which is a significantly lower frequency than the weekly applications commonly used in practice for both calcific RCRSP and other tendinopathies, such as the Achilles tendon [15,65]. Other stimulation therapies also employ a higher treatment frequency, often involving multiple sessions per week, as shown in Table 2. An umbrella review of systematic reviews and meta-analyses also indicated that the effects of ESWT on tendinopathies (in general) are effective. In some cases, long-term follow-up examinations also showed lasting therapeutic effects that were greater than those seen in the US control group [66]. Furthermore, there is a high potential for bias in the study by Speed et al. [56], especially because of incomplete or missing outcome data.

The systematic review and meta-analysis of Xiong et al. [42], for example, examined the use of ESWT for both calcific and non-calcific rotator cuff tendinitis and found that low-energy ESWT did not significantly reduce pain. The study by Speed et al. [56] was also included in this review and utilized low-energy ESWT. Previous reviews indicated that high-energy ESWT reduced pain in calcific rotator cuff tendinitis [42]. Based on these findings, it is recommended that future research also investigates high-energy ESWT for non-calcific rotator cuff tendinitis.

The currently available data do not provide consistent results regarding the effects of ESWT at the end of treatment or during follow-up in non-calcifying RCRSP. Therefore, there is an urgent need for methodologically high-quality studies with a lower potential for bias, larger sample sizes, clearly defined ESWT protocols, and an appropriate treatment frequency, in order to validly assess the efficacy of this therapy.

### 4.2. PBM

The aggregated data on the VAS and SPADI suggest that PBM has a positive effect on pain, with a weighted mean change of 0.43 cm (95% CI: 0.31 to 0.55) for VAS [8,16,33,34,35,36,37,38,39] and 3.99% (95% CI: 0.08 to 7.9) for the SPADI [8,16,33,37,38]. Since the CIs for both variables showed exclusively positive values, PBM can be assumed to be statistically effective, albeit clinical effects can be debated. Subgroup analysis revealed differences between the types of intervention (LLLT and HPLT). HPLT achieved consistent end-of-treatment effects in all the included studies (VAS-weighted mean change = 2.21 cm; 95% CI: 1.16 to 3.26) [33,34,37], while the evidence for LLLT varied greatly: three studies reported benefits in favor of LLLT [8,35,36], while three others showed better results in the control groups [16,38,39] (VAS-weighted mean change = −0.23 cm; 95% CI: −0.42 to −0.04). When compared with the literature that (co-) treated other structures in the shoulder, a very heterogeneous picture emerges here as well [67,68]. The applied dose may account for the heterogeneity observed in the LLLT group. The World Association for Photobiomodulation Therapy recommends minimum dosages of 4 J per point at 780 to 860 nm, and 2 J per point at 904 nm [69]. These thresholds were often not met or were only marginally achieved. Because they represent minimum values, the applied dose may have been insufficient to elicit a clear mean effect, and higher doses might have produced larger effects. Alternatively, the findings may reflect subgroup-specific treatment effects [38], random measurement variability (i.e., random error), and/or natural fluctuations in pain.

While PBM [16,32,34] showed favorable follow-up effects in VAS and SPADI data across nearly all studies, it is possible that these outcomes stem from an interaction with other therapies administered alongside PBM (VAS-weighted mean change = 1.13 cm, 95% CI: −0.29 to 2.55). However, due to the small number of studies, these results should be interpreted with caution. For example, when looking at the same intervention in the context of the end-of-treatment effect, it becomes evident that a different picture emerges when a larger body of data are available. It is also possible in this case that the HPLT subgroup provided consistent results, whereas the LLLT subgroup showed inconsistent outcomes.

De la Barra Ortiz et al. [70] showed in a systematic review and meta-analysis that HPLT is superior to US in terms of pain relief at the end of treatment in an intra-study comparison. Our systematic review, which primarily considered a cross-study comparison, showed a trend in the same direction: HPLT achieved a weighted mean VAS change of 2.18 cm (95% CI: 1.03 to 3.33), while US achieved a change of 0.72 cm (95% CI: 0.46 to 0.98) [71]. Furthermore, HPLT achieved the minimal clinically important difference (MCID) of 1.4 cm on the VAS, which underscores the clinical relevance of the results. The study designs of the included PBM studies do not allow for a separation of the main effect of PBM from a potential interaction effect with conservative protocols.

### 4.3. US

US in combination with conservative measures showed consistent end-of-treatment and follow-up effects on pain reduction (VAS-weighted mean change = 0.72 cm, 95% CI: 0.46 to 0.98; weighted mean change = 0.88 cm, 95% CI: 0.67 to 1.09). In all the included studies [32,35,40,51,52,53], US was superior to the control group, with the measured effects confirmed on the VAS and by other instruments such as the NHP, SF36, and CMS. Furthermore, all the CIs were in the positive range. This consistency suggests that the end-of-treatment efficacy of US is robust, with robust follow-up effects also detectable after completion of therapy in individual studies. Given that ultrasound was combined with other conservative protocols, we cannot rule out whether the results reflect a main effect, an interaction effect, or a simultaneous contribution of both factors.

All studies utilized a frequency of 1 MHz [32,40,51,52,53], with the exception of Calis et al., who employed 3 MHz [35]. Since the subacromial space and rotator cuff tendons are located at a depth of over 2.5 cm, a frequency of 1 MHz is appropriate if the goal is to achieve an effect at this depth [72]. To achieve thermal stimulation, a tissue temperature of 43 °C should be reached [72]. However, the parameters used in the studies [32,51,52]—i.e., 5 min at 1 W intensity—do not appear to be sufficient to generate this temperature increase [72]. Sen et al. [32] even used pulsed US, whose short application of 5 min also probably did not lead to a temperature increase to 43 °C [72]. Despite this, all the interventions reported pain relief, suggesting that pain reduction may be possible, even without relevant thermal effects.

In the study by Manca et al. [73] on myofascial trigger points, sham US appeared sufficient to produce a hypoalgesic effect. In contrast, in the studies by García et al. [52] and Kelle et al. [51], the control groups also received a sham US, yet a hypoalgesic effect of active US was still observed. This suggests that the effects reported in these studies can be attributed to the specific therapeutic action of US and not to placebo-related effects. However, it is possible that a placebo effect in the other intervention groups may have reinforced the observed pain relief effect, which could lead to an overestimation of the pure US effect. It should also be noted that the sham US groups did not necessarily have to show a hypoalgesic effect; therefore, the results should be interpreted with caution [74]. Although positive trends were observed for US, treatment effects remain uncertain and may be minimal or non-existent depending on the patient subgroup.

### 4.4. ET

Almost all outcome measures across the studies indicated that ET has an end-of-treatment effect on pain (VAS-weighted mean change = 1.55 cm, 95% CI: 0.41 to 2.69) [18,40,41,52,55]. It is important to consider that the observed effect of ET could be attributed to either a main effect, an interaction effect, or a combination of both. The only exception was Koumantakis et al. [54], who used the SPADI exclusively and reported an opposite effect. However, their sample size was small, with only nine participants in the ET group and eight in the control group. In addition, the treatment frequency was considerably lower—twice per week initially and once per week later—compared to the other studies, which applied three to five sessions per week [18,40,41,52,55]. These aspects may explain the contradictory end-of-treatment effect observed by Koumantakis et al. [54].

In this review, various ET modalities were aggregated under the overarching term ET. The included studies evaluated TENS, interferential current therapy, iontophoresis, and transfer of electricity for capacitive and resistive tissues (TECAR). It is critical to acknowledge that while these modalities differ substantially in their underlying biophysical mechanisms, they consistently demonstrated a positive end-of-treatment effect across the respective trials.

Regarding the follow-up effect on pain, almost every study, regardless of the measurement instrument used, reported a reduction in pain [40,41,52]. These results indicate a trend towards ET. Nazligul et al. [41] reported an effect for the ET group and the control group, which varied according to the measurement instrument used. This highlights an issue regarding the reliability of the assessment tools. More research is also needed here to gain more certainty about the follow-up effects.

The observed reduction in pain via ET is consistent with results from other animal and human studies that have demonstrated an analgesic effect of ET (TENS). Nevertheless, this hypoalgesic effect decreases over days when using identical stimulation parameters with increasing treatment duration [75,76]. This phenomenon can be explained by the fact that ET application triggers the release of endogenous substances that desensitize opioid receptors [75]. Based on these findings, no effect should be expected after the measurement time points (weeks and months) of the included studies. However, the majority of the included studies did not feature TENS or did not feature TENS exclusively. The study by Rani et al. [55] applied TENS over five consecutive days and observed significant pain relief. After five days, the adaptation of the opioid receptor and, consequently, the hypoalgesic effect can be expected to have diminished [75,76].

Having addressed these central mechanisms of pain modulation, the question arises as to how local processes contribute to the pathology and how they might be targeted through therapeutic interventions. Subacromial impingement syndrome is part of the clinical picture of RCRSP [2]. Neer [77] described that this condition involves physical contact between the coracoacromial ligament and the anterior third of the acromion with some of the underlying structures. The extent to which this mechanism is responsible as a cause for the development of RCRSP is currently unclear [1]. The study by Beard et al. [78] showed that subacromial decompression is not superior to simple arthroscopy after 12 months. This is despite the fact that the alleged cause was eliminated. Furthermore, the tear sites were at a different location than where the impingement can occur [2]. However, there is also information that suggests the involvement of impingement. Impingement syndrome occurs more frequently on the symptomatic shoulder than on the symptom-free shoulder [79]. In addition, individuals with a degenerative supraspinatus tear have a smaller impingement-free range of motion and critical shoulder angle and greater tuberosity angle [80]. It has also been reported that the subacromial bursa can become entrapped under the coracoacromial ligament [81]. Overall, this suggests that impingement should be considered less as a primary cause and more as a contributing factor in the etiology and maintenance of RCRSP. Injecting a local anesthetic into the subacromial space leads to a significant reduction in pain [38,41,82]. Substances such as substance P and vascular endothelial growth factor in the subacromial bursa are involved in pain perception [17]. This indicates that the subacromial bursa contributes to pain perception. Human and animal studies suggest that the bursa could even contribute to the healing of supraspinatus tendinopathy [83,84]. It might be that mechanical irritation, i.e., impingement, changes the bursa [84]. An animal study demonstrated that it is also possible that the tendinopathy stimulates the bursa [83]. None of the device-assisted modalities would be primarily capable of reducing impingement syndrome. If so, it would be indirectly through pain reduction, which secondarily leads to improved function. Beyrami et al. [33] were able to reduce subacromial fluid with HPLT. By placing the laser head on the subacromial space, it might thus be possible to intervene positively on the bursa. Due to the potential influence of the bursa on supraspinatus tendon healing [83,84], it is also conceivable that device-assisted modalities could have an indirect effect on tendinopathy. Accordingly, the bursa might be stimulated, leading to the production of substances that act in a paracrine manner to support the healing of the supraspinatus tendon. If this assumption is correct, the application of, for example, high-power laser therapy should be directed at the subacromial space and should be implemented in this way in future treatments of supraspinatus tendinopathy. In addition, ESWT, PBM, and US can primarily modify the structurally altered tendons [21,22,23,24,25]. Since exercise therapy was almost always performed additionally in the studies, the potential impingement could also have been positively influenced by this [85]. The bony morphology was, of course, not altered. It has been shown that central sensitization also occurs in RCRSP [86]. This would be another area in which the device-assisted modalities could intervene.

Regarding adverse effects, half of the studies provided information, while the other half did not report on this aspect. Underreporting of adverse effects is a phenomenon observed with other clinical conditions as well [87]. No study identified adverse effects. With the exception of the study by Speed et al. [56], in which one participant did not tolerate ESWT, as no further details were provided, a causal relationship with ESWT cannot be clearly assessed, although a connection appears possible. Given the incomplete reporting of adverse events across the included studies, definitive conclusions regarding safety cannot be drawn; however, no serious adverse events were reported in the studies that provided safety data. The main costs associated with these therapies are more likely to lie in terms of time and social burden [88]. Future studies must ensure that treatment safety is reported [87]. This enables a complete cost–benefit evaluation of a treatment.

When analyzing the pain data, it becomes evident that RCRSP represents a chronic condition (maybe for several months or up to one or two years). Their pain occurred during activity, at rest, or at night. This persisted, despite the fact that participants had received multiple conservative physiotherapeutic treatments, such as stretching and strengthening exercises. Moreover, these follow-up effect data also reflect the natural course of the condition and are confirmed by a study with a no-intervention group [78]. As mentioned previously, the MCID was 1.4 cm on the VAS. Only the end-of-treatment effects for the PBM subgroup HPLT and the ET group achieved this value [71]. However, in light of this persistent pain burden across different domains, even a therapy that yields only a modest effect can be regarded as beneficial. Through a process of shared decision-making, patients should evaluate whether an incremental benefit justifies the associated trade-offs, such as time burden and financial costs. Chronic pain remains a factor in RCRSP, irrespective of the intervention used (also a large array simultaneously). Furthermore, incorporating device-assisted modalities does not eliminate the pain entirely. Other methods, such as scapular stabilization training, are similarly unable to fully resolve the pain, even when they improve function beyond what stretching and strength training can already achieve [89]. This indicates that further research is necessary to find one or a few physiotherapeutic interventions that demonstrate a large effect.

All intervention groups, with the exception of Speed et al. [56], received supplementary interventions. Stretching and strength training were very common among these interventions. The data from the control groups revealed that some pain relief occurs over time. This finding was also confirmed by a meta-analysis and an umbrella review of systematic reviews and meta-analyses [13,66]. No monotherapy effects were measured in this study; instead, combined effects were assessed. Accordingly, the device-assisted modalities, if effective, provide an additive effect. What would also be interesting here is how the effect is on RCRSP as a monotherapy, or also when multiple device-assisted modalities are combined. Beyrami et al. [33] found that the combination of PBM (HPLT), US, ET, hot pack, and exercise therapies was superior to the group without PBM. This, too, did not yet result in complete pain relief within the groups. Furthermore, recent evidence (e.g., Manocchio et al. [90]) underscores that RCRSP is best managed within individualized, multimodal frameworks, where passive modalities serve as complementary, additive components alongside active rehabilitation rather than standalone solutions.

An examination of the intervention frequency reveals that therapy is conducted almost exclusively three to five times per week. For example, when looking at the intervention duration of US, it is evident that the intervention duration is usually very short. However, the frequency of three to five sessions per week represents a relatively high time demand, assuming that the therapy was not self-administered. In clinical practice, this could lead to difficulties for working individuals in adhering to these parameters. ESWT is the exception here.

### 4.5. Limitations

Among the included studies were papers that insufficiently described their diagnostic criteria. Consequently, there is uncertainty regarding the suitability of these studies for our RCRSP diagnosis. Pekyavas and Baltaci [37] stated that the participants were diagnosed by a “physical medicine and rehabilitation doctor” with no further details. A reference to authority (appeal to authority) should not be considered sufficient. Rani et al. [55] and Pavlovskiy et al. [50] reported that they included participants with rotator cuff disease and shoulder pain syndrome. However, they did not provide any information regarding the diagnostic procedure. Consequently, the available data remain somewhat incomplete. The diagnostic methods need to be transparently detailed so that an external reader can fully comprehend the study’s content and to facilitate the study’s reproduction.

The inclusion and exclusion procedures varied considerably across the studies. Only some studies used MRI or ultrasound to rule out calcification, acromioclavicular arthritis, glenohumeral arthritis, and full-thickness tears [8,16,18,32,35,39,40,41,51,53]. Therefore, it could only be ensured for a portion of the patients included in this systematic review that they met the eligibility criteria of the present study. Future RCTs should ensure that MRI or ultrasound is performed to avoid errors during the inclusion and exclusion process.

Numerous studies had to be excluded because they included participants who had received prior treatment within three months before enrollment. For future studies, it is therefore important that the washout period (the time between the last intervention and the study entry) is not too short in order to rule out crossover effects.

In the context of the present findings, it is important to note that, although the included interventions were predominantly evaluated as standalone modalities, their effects should be interpreted from a cumulative perspective. This is particularly relevant given that such treatments are frequently combined in clinical practice. Therefore, the reported outcomes could reflect an additive effect or an interaction between the therapies.

A further limitation is the heterogeneity of the adjunct conservative treatments provided to the intervention (and control) group. This variability potentially leads to more diverse outcomes, as an effective baseline conservative therapy may leave less room for device-assisted modalities to demonstrate incremental pain reduction (a potential ‘ceiling effect’). Furthermore, different combinations of treatments may result in varying interaction effects.

Regarding a cost–benefit analysis, it would be of particular interest to determine the efficacy of these modalities when applied as a standalone treatment. A fundamental aspect of RCRSP is that it represents a clinical entity characterized by inherent diagnostic uncertainty regarding the specific pain-generating structures [1]. From this perspective, the application of device-assisted modalities is often a process of approximating the symptomatic area rather than a pinpoint-targeted intervention.

A limitation of the currently available body of evidence is that the nature of primary studies precluded a formal meta-analysis. This was dictated by significant methodological challenges within the identified literature, which predominantly utilized ordinal scales and questionnaires to assess pain. This reliance on varied measurement tools introduces substantial variability that fundamentally undermines the validity of any aggregated results. Attempting statistical pooling under these circumstances would risk producing misleading conclusions. Consequently, a systematic approach was maintained to ensure a transparent analysis without the pitfalls inherent in a premature meta-analysis.

## 5. Conclusions

Regarding the end-of-treatment effects, HPLT, US, and ET consistently demonstrated improvements across the studies. Furthermore, US showed an albeit modest but robust follow-up effect across all studies. For PBM, a potential follow-up effect was observed. However, this must be viewed with caution because no subgroup analysis could be conducted. An effect might also only be detectable in the HPLT subgroup here. The device-assisted modalities were almost always combined with other therapeutic approaches (no monotherapies) and compared against other treatment forms, thereby showing an additive effect. Further high-quality RCTs are needed for ESWT to allow for robust conclusions regarding its effectiveness. A large proportion of the existing ESWT studies have investigated calcific rotator cuff tendinopathy, which has a different pathophysiology than the non-calcific form and must therefore be studied separately. Additional studies with follow-up effect assessments are also needed for ET and the PBM subgroups LLLT and HPLT. Given that RCRSP is a chronic pain condition, the therapies presented here are recommended, even if they only have modest additive effects. HPLT applied to the subacromial bursa and the tendons tends to be an effective treatment modality. Although the included interventions were analyzed predominantly as single modalities, the interpretation of the findings should be considered in a broader, cumulative context. It should be acknowledged that, due to the study design, the observed effects must be attributed to either the device-assisted modalities alone or an interaction effect. This consideration, together with the heterogeneity of the included studies, the limited number of trials for certain modalities, and methodological variability, calls for a cautious interpretation of some of the results.

## Figures and Tables

**Figure 1:**
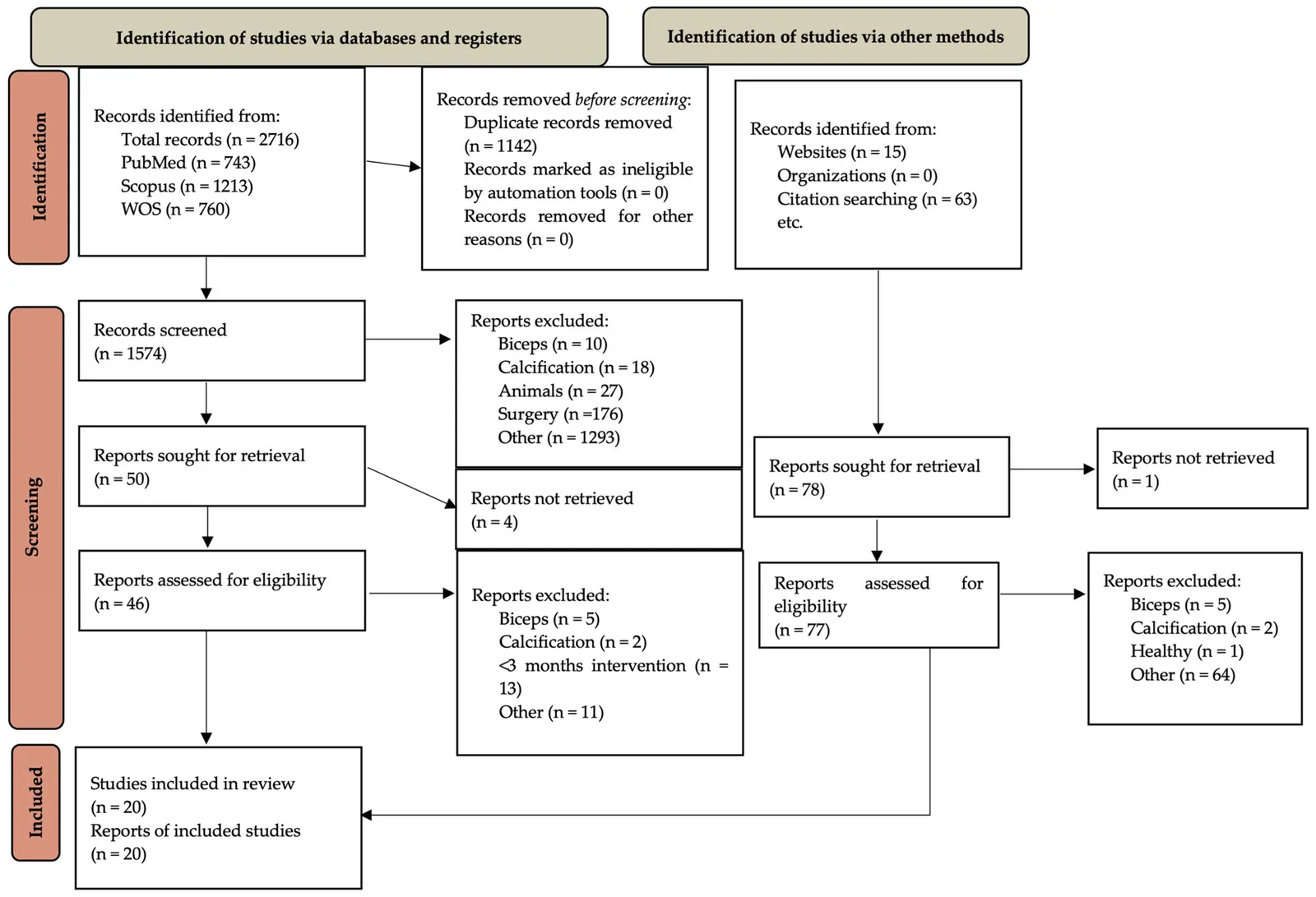
Flow diagram of the study selection process [49].

**Figure 2:**
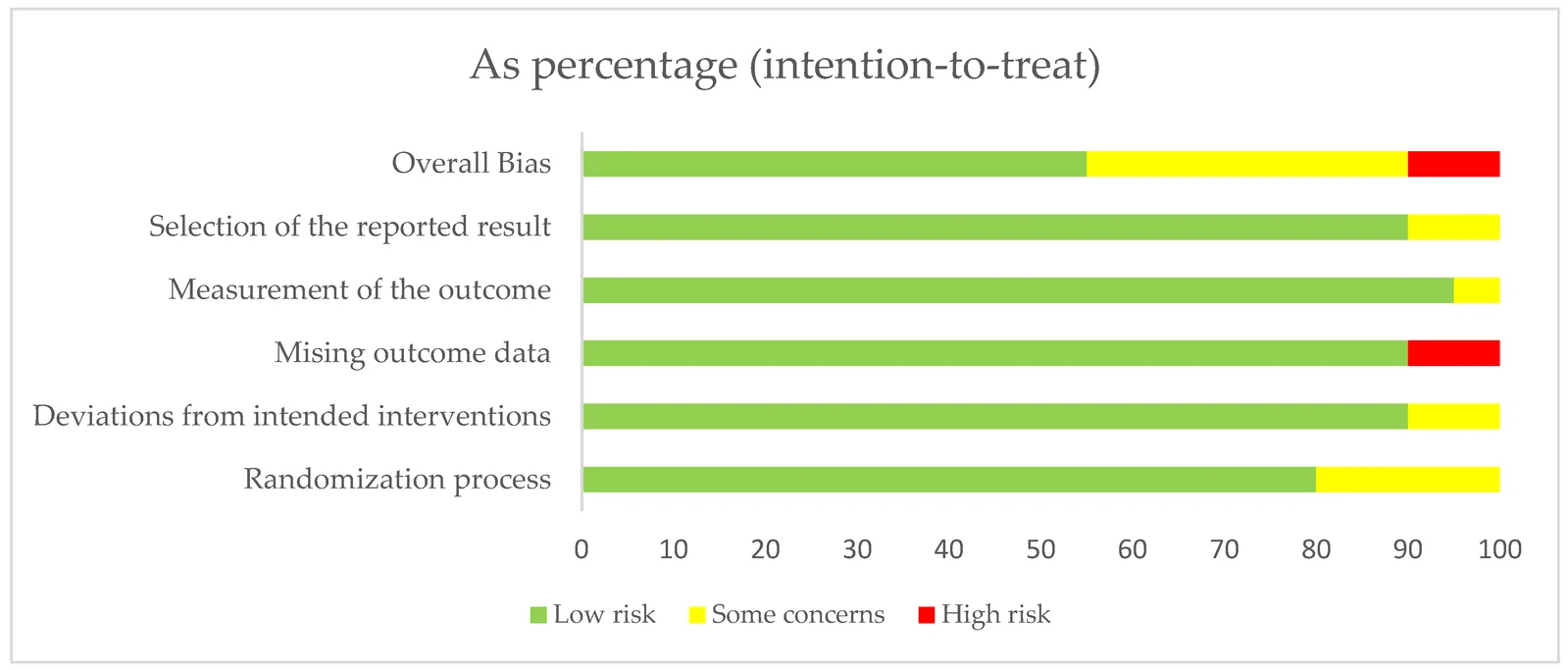
Risk–of–bias graph.

**Figure 3:**
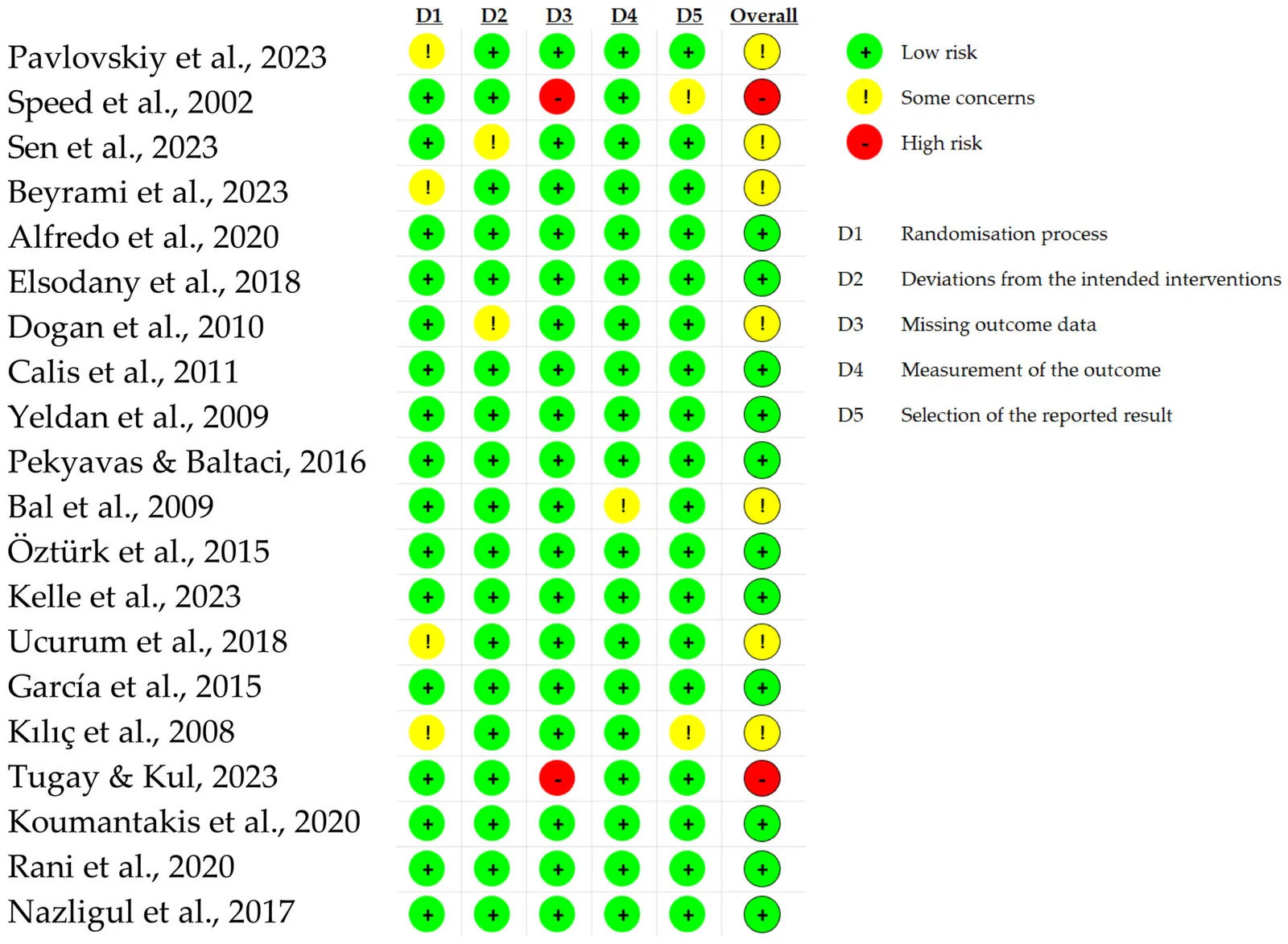
Risk–of–bias summary [8,16,18,32,33,34,35,36,37,38,39,40,41,50,51,52,53,54,55,56].

**Table 1:** Description of the outcome variables.

Outcome	Scoring System	Value Range
VAS	10 cm line	0 = no pain, 10 = most imaginable pain
SPADI–pain	Subscale: 5 items from 0 to 10 points	0% = lowest value, 100% = highest value
CMS–pain	Subscale: 15 points	0 = no pain, 15 = intolerable pain
NRS	0 to 10 points	0 = no pain, 10 = most imaginable pain
SF36	Subscale: 1 to 6 points bodily pain, 1 to 5 points pain interference	0 = maximum pain burden, 100 = no interference
NHP–pain	Subscale: 8 questions	0 = maximum pain burden, 100 = no interference

VAS = visual analog scale, SPADI–pain = Shoulder Pain and Disability Index–pain, NRS = numerical rating scale, CMS–pain = Constant-Murley score–pain, SF36–bodily pain = Short Form 36–bodily pain, NHP = Nottingham Health Profile–pain.

**Table 3:** Summary of studies reporting end-of-treatment and follow-up effects of ESWT, PBM, US, and ET on pain (difference to controls).

Study	Parameters	Difference to Control End-of-Treatment Effects (cm/Points)	Difference to Control Follow-Up Effects (cm/Points)
**ESWT**			
Pavlovskiy et al. [50]	VAS	0.50	
Speed et al. [56]	VAS		−0.08
**PBM**			
Sen et al. [32]	VAS SPADI–pain		0.30 2.51
Beyrami et al. [33]	VAS	0.20	
	SPADI–pain	1.72	
Alfredo et al. [16]	NRS	−0.23	−0.28
	SPADI–pain	4.96	15.82
Elsodany et al. [34]	VAS	0.31	0.86
Dogan et al. [8]	VAS	0.13	
	SPADI–pain	−0.2	
Calis et al. [35]	VAS	0.06	
Yeldan et al. [36]	VAS	0.02	
	CMS–pain	0.44	
Pekyavas and Baltaci [37]	VAS	0.06	
	SPADI–pain	1.23	
Bal et al. [38]	VAS	−0.07	0.25
	SPADI–pain	−3.72	
Öztürk et al. [39]	VAS	−0.06	
**US**			
Sen et al. [32]	VAS SPADI–pain		0.2 6.4
Kelle et al. [51]	VAS	0.09	0.1
	NHP–pain	9.1	4.00
Ucurum et al. [40]	VAS	0.02	0.10
García et al. [52]	CMS–pain		−3.10
	SF36–bodily pain		−13.50
Calis et al. [35]	VAS	0.39	
Kılıç et al. [53]	VAS	0.22	0.41
**ET**			
Tugay and Kul [18]	VAS	0.48	
Koumantakis et al. [54]	SPADI–pain	−3.70	
Ucurum et al. [40]	VAS	0.08	0.02
García et al. [52]	CMS–pain		−1.00
	SF36–bodily pain		−7.60
Rani et al. [55]	SPADI–pain	35.33	
Nazligul et al. [41]	VAS	0.99	2.45
	CMS–pain	−0.5	0.5

Positive VAS, NRS, SPADI–pain, and NHP values indicate that pain decreased in the intervention group, and vice versa. Negative CMS–pain and SF36 values indicate that pain decreased in the intervention group, and vice versa. VAS = visual analog scale, SPADI–pain = Shoulder Pain and Disability Index–pain, NRS = numerical rating scale, CMS–pain = Constant-Murley score–pain, SF36–bodily pain = Short Form 36–bodily pain, NHP = Nottingham Health Profile–pain (Safety).

## Data Availability

No new data were created or analyzed in this study. Data sharing is not applicable to this article.

## Abbreviations

CG	Control group
CMS	Constant-Murley score
d	Days/s
ESWT	Extracorporeal shock wave therapy
ET	Electrotherapy modalities
Grp	Group
HPLT	High-power laser therapy
IG	Intervention group
LLLT	Low-level laser therapy
m	Month/s
MCID	Minimal clinically important difference
MRI	Magnetic resonance imaging
NHP	Nottingham Health Profile
NR	Not reported
NRS	Numerical rating scale
OT	Other treatments
PBM	Photobiomodulation
RCRSP	Rotator cuff related shoulder pain
SF36	Short Form 36
SPADI	Shoulder Pain and Disability Index
TENS	Transcutaneous electrical nerve stimulation
US	Ultrasound
VAS	Visual analog scale
w	Week/s

## References

[1] Requejo-Salinas N., Lewis J., Michener L.A., La Touche R., Fernández-Matías R., Tercero-Lucas J., Camargo P.R., Bateman M., Struyf F., Roy J.-S. (2022). International physical therapists consensus on clinical descriptors for diagnosing rotator cuff related shoulder pain: A Delphi study. Braz. J. Phys. Ther..

[2] Lewis J. (2016). Rotator cuff related shoulder pain: Assessment, management and uncertainties. Man. Ther..

[3] Sansone V., Maiorano E., Galluzzo A., Pascale V. (2018). Calcific tendinopathy of the shoulder: Clinical perspectives into the mechanisms, pathogenesis, and treatment. Orthop. Res. Rev..

[4] Chester R., Jerosch-Herold C., Lewis J., Shepstone L. (2016). Psychological factors are associated with the outcome of physiotherapy for people with shoulder pain: A multicentre longitudinal cohort study. Br. J. Sports Med..

[5] Sachinis N.P., Yiannakopoulos C.K., Chalidis B., Kitridis D., Givissis P. (2022). Biomolecules Related to Rotator Cuff Pain: A Scoping Review. Biomolecules.

[6] Lo C.N., Leung B.P.L., Sanders G., Li M.W.M., Ngai S.P.C. (2022). The major pain source of rotator cuff-related shoulder pain: A narrative review on current evidence. Musculoskelet. Care.

[7] Seitz A.L., McClure P.W., Finucane S., Boardman N.D., Michener L.A. (2011). Mechanisms of rotator cuff tendinopathy: Intrinsic, extrinsic, or both?. Clin. Biomech..

[8] Dogan S.K., Ay S., Evcik D. (2010). The effectiveness of low laser therapy in subacromial impingement syndrome: A randomized placebo controlled double-blind prospective study. Clinics.

[9] Leong H., Fu S., He X., Oh J., Yamamoto N., Yung S. (2019). Risk factors for rotator cuff tendinopathy: A systematic review and meta-analysis. J. Rehabil. Med..

[10] Hopkins C., Fu S.-C., Chua E., Hu X., Rolf C., Mattila V.M., Qin L., Yung P.S.-H., Chan K.-M. (2016). Critical review on the socio-economic impact of tendinopathy. Asia-Pac. J. Sports Med. Arthrosc. Rehabil. Technol..

[11] Cho C.-H., Jung S.-W., Park J.-Y., Song K.-S., Yu K.-I. (2013). Is shoulder pain for three months or longer correlated with depression, anxiety, and sleep disturbance?. J. Shoulder Elb. Surg..

[12] Powell J.K., Lewis J., Schram B., Hing W. (2024). Is exercise therapy the *right* treatment for rotator cuff-related shoulder pain? Uncertainties, theory, and practice. Musculoskelet. Care.

[13] Wu D., Wen Z., Ke H., Zhang J., Zhong S., Teng J., Xu L., Li J., Shao Y., Zeng C. (2025). Specific modes of exercise to improve rotator cuff-related shoulder pain: Systematic review and meta-analysis. Front. Bioeng. Biotechnol..

[14] Boudreault J., Desmeules F., Roy J., Dionne C., Frémont P., MacDermid J. (2014). The efficacy of oral non-steroidal anti-inflammatory drugs for rotator cuff tendinopathy: A systematic review and meta-analysis. J. Rehabil. Med..

[15] Xue X., Song Q., Yang X., Kuati A., Fu H., Liu Y., Cui G. (2024). Effect of extracorporeal shockwave therapy for rotator cuff tendinopathy: A systematic review and meta-analysis. BMC Musculoskelet. Disord..

[16] Alfredo P.P., Bjordal J.M., Junior W.S., Marques A.P., Casarotto R.A. (2020). Efficacy of low-level laser therapy combined with exercise for subacromial impingement syndrome: A randomised controlled trial. Clin. Rehabil..

[17] Martins J.P.S., de Lima C.J., Fernandes A.B., Alves L.P., Neto O.P., Villaverde A.B. (2022). Analysis of pain relief and functional recovery in patients with rotator cuff tendinopathy through therapeutic ultrasound and photobiomodulation therapy: A comparative study. Lasers Med. Sci..

[18] Tugay M., Kul A. (2023). Efficacy of Interferential Current Therapy in Patients Diagnosed with Subacromial Impingement Syndrome. Eurasian J. Med..

[19] Svendsen S.W., Gelineck J., Mathiassen S.E., Bonde J.P., Frich L.H., Stengaard-Pedersen K., Egund N. (2004). Work above shoulder level and degenerative alterations of the rotator cuff tendons: A magnetic resonance imaging study. Arthritis Rheum..

[20] Jo C.H., Shin W.H., Park J.W., Shin J.S., Kim J.E. (2016). Degree of tendon degeneration and stage of rotator cuff disease. Knee Surg. Sports Traumatol. Arthrosc..

[21] Farcic T.S., Baldan C.S., Cattapan C.G., Parizotto N.A., Joao S.M.A., Casarotto R.A. (2013). Treatment time of ultrasound therapy interferes with the organization of collagen fibers in rat tendons. Braz. J. Phys. Ther..

[22] Yeung C.K., Guo X., Ng Y.F. (2005). Pulsed ultrasound treatment accelerates the repair of Achilles tendon rupture in rats. J. Orthop. Res..

[23] Wood V.T., Pinfildi C.E., Neves M.A., Parizoto N.A., Hochman B., Ferreira L.M. (2010). Collagen changes and realignment induced by low-level laser therapy and low-intensity ultrasound in the calcaneal tendon. Lasers Surg. Med..

[24] Mussttaf R.A., Jenkins D.F.L., Jha A.N. (2019). Assessing the impact of low level laser therapy (LLLT) on biological systems: A review. Int. J. Radiat. Biol..

[25] Vetrano M., D’aLessandro F., Torrisi M.R., Ferretti A., Vulpiani M.C., Visco V. (2011). Extracorporeal shock wave therapy promotes cell proliferation and collagen synthesis of primary cultured human tenocytes. Knee Surg. Sports Traumatol. Arthrosc..

[26] Chen M.-H., Huang Y.-C., Sun J.-S., Chao Y.-H., Chen M.-H. (2014). Second messengers mediating the proliferation and collagen synthesis of tenocytes induced by low-level laser irradiation. Lasers Med. Sci..

[27] Ogden J.A., Tóth-Kischkat A., Schultheiss R. (2001). Principles of Shock Wave Therapy. Clin. Orthop. Relat. Res..

[28] Lawrence J., Sorra K. (2024). Photobiomodulation as Medicine: Low-Level Laser Therapy (LLLT) for Acute Tissue Injury or Sport Performance Recovery. J. Funct. Morphol. Kinesiol..

[29] Penberthy W.T., Vorwaller C.E. (2021). Utilization of the 1064 nm Wavelength in Photobiomodulation: A Systematic Review and Meta-Analysis. J. Lasers Med. Sci..

[30] Fuentes J.P., Olivo S.A., Magee D.J., Gross D.P. (2010). Effectiveness of Interferential Current Therapy in the Management of Musculoskeletal Pain: A Systematic Review and Meta-Analysis. Phys. Ther..

[31] Johns L.D. (2002). Nonthermal Effects of Therapeutic Ultrasound: The Frequency Resonance HypothesisNonthermal Effects of Therapeutic Ultrasound: The Frequency Resonance Hypothesis. J. Athl. Train..

[32] Sen E.I., Arman S., Tseveendorj N., Yılmaz E., Oral A., Capan N. (2023). Low-level laser therapy versus ultrasound therapy combined with home-based exercise in patients with subacromial impingement syndrome: A randomized-controlled trial. Turk. J. Phys. Med. Rehabil..

[33] Beyrami M., Soltani A., Eftekharsadat B., Sarbakhsh P., Habibi M., Oskouei A.E. (2023). Effects of High Power Laser Therapy on Clinical and Sonographic Findings in People with Chronic Rotator Cuff Tendinitis. J. Adv. Med. Biomed. Res..

[34] Elsodany A.M., Alayat M.S.M., Ali M.M.E., Khaprani H.M. (2018). Long-Term Effect of Pulsed Nd:YAG Laser in the Treatment of Patients with Rotator Cuff Tendinopathy: A Randomized Controlled Trial. Photomed. Laser Surg..

[35] Calis H.T., Berberoglu N., Calis M. (2011). Are ultrasound, laser and exercise superior to each other in the treatment of subacromial impingement syndrome? A randomized clinical trial. Eur. J. Phys. Rehabil. Med..

[36] Yeldan I., Cetin E., Ozdincler A.R. (2009). The effectiveness of low-level laser therapy on shoulder function in subacromial impingement syndrome. Disabil. Rehabil..

[37] Pekyavas N.O., Baltaci G. (2016). Short-term effects of high-intensity laser therapy, manual therapy, and Kinesio taping in patients with subacromial impingement syndrome. Lasers Med. Sci..

[38] Bal A., Eksioglu E., Gurcay E., Gulec B., Karaahmet O., Cakci A. (2009). Low-Level Laser Therapy in Subacromial Impingement Syndrome. Photomed. Laser Surg..

[39] Öztürk P.A., Altınay İ.Ş., Karatepe G., Kaya T., Günaydın R. (2015). Effectiveness of low-level laser therapy in patients with subacromial impingement syndrome: A randomized, placebo controlled, prospective study. J. Tepecik Educ. Res. Hosp..

[40] Ucurum S.G., Kaya D.O., Kayali Y., Askin A., Tekindal M.A. (2018). Comparison of different electrotherapy methods and exercise therapy in shoulder impingement syndrome: A prospective randomized controlled trial. Acta Orthop. Traumatol. Turc..

[41] Nazligul T., Akpinar P., Aktas I., Ozkan F.U., Hartevioglu H.C. (2018). The effect of interferential current therapy on patients with subacromial impingement syndrome: A randomized, double-blind, sham-controlled study. Eur. J. Phys. Rehabil. Med..

[42] Xiong Y., Wen T., Jin S., Lin L., Shao Q., Peng Y., Zheng Q., Li W. (2024). Efficacy and safety of extracorporeal shock wave therapy for upper limb tendonitis: A systematic review and meta-analysis of randomized controlled trials. Front. Med..

[43] Hao J., Yao Y., Remis A., Sun Y., Zhu D., Wu S. (2024). Effects of high-intensity laser therapy on subacromial impingement syndrome: A systematic review and meta-analysis. Lasers Med. Sci..

[44] Kamonseki D.H., da Rocha G.M., Ferreira V.M.L.M., Ocarino J.d.M., Pogetti L.S. (2023). Extracorporeal Shockwave Therapy for the Treatment of Noncalcific Rotator Cuff Tendinopathy. Am. J. Phys. Med. Rehabil..

[45] Liberati A., Altman D.G., Tetzlaff J., Mulrow C., Gøtzsche P.C., Ioannidis J.P., Clarke M., Devereaux P.J., Kleijnen J., Moher D. (2009). The PRISMA statement for reporting systematic reviews and meta-analyses of studies that evaluate healthcare interventions: Explanation and elaboration. J. Clin. Epidemiol..

[46] Hozo S.P., Djulbegovic B., Hozo I. (2005). Estimating the mean and variance from the median, range, and the size of a sample. BMC Med. Res. Methodol..

[47] Wan X., Wang W., Liu J., Tong T. (2014). Estimating the sample mean and standard deviation from the sample size, median, range and/or interquartile range. BMC Med. Res. Methodol..

[48] Sterne J.A.C., Savović J., Page M.J., Elbers R.G., Blencowe N.S., Boutron I., Cates C.J., Cheng H.Y., Corbett M.S., Eldridge S.M. (2019). RoB 2: A revised tool for assessing risk of bias in randomised trials. BMJ.

[49] Page M.J., McKenzie J.E., Bossuyt P.M., Boutron I., Hoffmann T.C., Mulrow C.D., Shamseer L., Tetzlaff J.M., Akl E.A., Brennan S.E. (2021). The PRISMA 2020 statement: An updated guideline for reporting systematic reviews. BMJ.

[50] Pavlovskiy S.A., Fesyun A.D., Konchugova T.V., Nikitin M.V., Kulchitskaya D.B. (2023). Effectiveness of Physiotherapy Methods in Medical Rehabilitation of Patients with Shoulder and Scapular Pain Syndrome in a Sanatorium: A Randomized Clinical Study. Bull. Rehabil. Med..

[51] Kelle B., Deniz V., Ortaç E.A. (2023). Phonophoresis treatment of subacromial impingement syndrome: Pulsed or continuous: A randomized-controlled clinical trial. Turk. J. Phys. Med. Rehabil..

[52] García I., Lobo C., López E., Serván J.L., Tenías J.M. (2015). Comparative effectiveness of ultrasonophoresis and iontophoresis in impingement syndrome: A double-blind, randomized, placebo controlled trial. Clin. Rehabil..

[53] Kılıç Ö., İçağasıoğlu A., Kolukısa Ş., Demirhan E., Aras H. (2008). Comparison of ultrasound and mobilisation in treatment of subacromial impingement syndrome. Medeni. Med. J..

[54] Koumantakis G., Samios D., Philippou A. (2020). Painful shoulder: The effect of corrective therapeutic exercise alone or in combination with manual therapy or electrotherapy. Arch. Hell. Med..

[55] Rani P., Kalyani V., Goyal T., Yadav R., Mishra R. (2020). Effect of transcutaneous electrical nerve stimulation therapy on pain and functional disability level among patients with rotator cuff disease-A randomized controlled trial. Int. J. Physiother..

[56] Speed C.A., Richards C., Nichols D., Burnet S., Wies J.T., Humphreys H., Hazleman B.L. (2002). Extracorporeal shock-wave therapy for tendonitis of the rotator cuff. J. Bone Jt. Surgery. Br. Vol..

[57] Schofer M.D., Hinrichs F., Peterlein C.D., Arendt M., Schmitt J. (2009). High- versus low-energy extracorporeal shock wave therapy of rotator cuff tendinopathy: A prospective, randomised, controlled study. Acta Orthop. Belg..

[58] Schmitt J., Tosch A., Hünerkopf M., Haake M. (2002). Die extrakorporale Stoßwellentherapie (ESWT) als therapeutische Option beim Supraspinatussehnen-syndrom?. Orthopade.

[59] Efe T., Felgentreff M., Heyse T.J., Stein T., Timmesfeld N., Schmitt J., Roessler P.P. (2014). Extracorporeal shock wave therapy for non-calcific supraspinatus tendinitis–10-year follow-up of a randomized placebo-controlled trial. Biomed. Eng./Biomed. Tech..

[60] Schmitt J., Haake M., Tosch A., Hildebrand R., Deike B., Griss P. (2001). Low-energy extracorporeal shock-wave treatment (ESWT) for tendinitis of the supraspinatus. J. Bone Jt. Surgery. Br. Vol..

[61] Ko J.-Y., Siu K.-K., Wang F.-S., Wang C.-J., Chou W.-Y., Huang C.-C., Kuo S.-J. (2020). The Therapeutic Effects of Extracorporeal Shock Wave Therapy (ESWT) on the Rotator Cuff Lesions with Shoulder Stiffness: A Prospective Randomized Study. BioMed Res. Int..

[62] Kvalvaag E., Roe C., Engebretsen K.B., Soberg H.L., Juel N.G., Bautz-Holter E., Sandvik L., Brox J.I. (2018). One year results of a randomized controlled trial on radial Extracorporeal Shock Wave Treatment, with predictors of pain, disability and return to work in patients with subacromial pain syndrome. Eur. J. Phys. Rehabil. Med..

[63] Li W., Zhang S.-X., Yang Q., Li B.-L., Meng Q.-G., Guo Z.-G. (2017). Effect of extracorporeal shock-wave therapy for treating patients with chronic rotator cuff tendonitis. Medicine.

[64] Li C., Li Z., Shi L., Wang P., Gao F., Sun W. (2021). Effectiveness of Focused Shockwave Therapy versus Radial Shockwave Therapy for Noncalcific Rotator Cuff Tendinopathies: A Randomized Clinical Trial. BioMed Res. Int..

[65] Paantjens M.A., Helmhout P.H., Backx F.J.G., van Etten-Jamaludin F.S., Bakker E.W.P. (2022). Extracorporeal Shockwave Therapy for Mid-portion and Insertional Achilles Tendinopathy: A Systematic Review of Randomized Controlled Trials. Sports Med.-Open.

[66] Girgis B., Duarte J.A. (2020). Physical therapy for tendinopathy: An umbrella review of systematic reviews and meta-analyses. Phys. Ther. Sport.

[67] Bingöl Ü., Altan L., Yurtkuran M. (2005). Low-Power Laser Treatment for Shoulder Pain. Photomed. Laser Surg..

[68] England S., Farrell A.J., Coppock J.S., Struthers G., Bacon P.A. (1989). Low Power Laser Therapy of Shoulder Tendonitis. Scand. J. Rheumatol..

[69] Dosage Recommendations. https://waltpbm.org/documentation-links/recommendations/.

[70] Ortiz H.A.d.l.B., Cortés A., Pizarro F., Sabaj N., Sainz E. (2025). Effectiveness of high-intensity laser therapy in the treatment of shoulder impingement syndrome: A systematic review and meta-analysis of randomised clinical trials. Physiother. Q..

[71] Tashjian R.Z., Deloach J., Porucznik C.A., Powell A.P. (2009). Minimal clinically important differences (MCID) and patient acceptable symptomatic state (PASS) for visual analog scales (VAS) measuring pain in patients treated for rotator cuff disease. J. Shoulder Elb. Surg..

[72] Draper D.O., Castel J.C., Castel D. (1995). Rate of Temperature Increase in Human Muscle During 1 MHz and 3 MHz Continuous Ultrasound. J. Orthop. Sports Phys. Ther..

[73] Manca A., Limonta E., Pilurzi G., Ginatempo F., De Natale E.R., Mercante B., Tolu E., Deriu F. (2014). Ultrasound and Laser as Stand-Alone Therapies for Myofascial Trigger Points: A Randomized, Double-Blind, Placebo-Controlled Study. Physiother. Res. Int..

[74] Ay S., Doğan Ş.K., Evcik D., Başer Ö.Ç. (2010). Comparison the efficacy of phonophoresis and ultrasound therapy in myofascial pain syndrome. Rheumatol. Int..

[75] Chandran P., Sluka K.A. (2003). Development of opioid tolerance with repeated transcutaneous electrical nerve stimulation administration. Pain.

[76] Liebano R.E., Rakel B., Vance C.G., Walsh D.M., Sluka K.A. (2011). An investigation of the development of analgesic tolerance to TENS in humans. Pain.

[77] Neer C.S. (1972). Anterior acromioplasty for the chronic impingement syndrome in the shoulder: A preliminary report. J. Bone Jt. Surg..

[78] Beard D.J., Rees J.L., Cook J.A., Rombach I., Cooper C., Merritt N., Shirkey B.A., Donovan J.L., Gwilym S., Savulescu J. (2018). Arthroscopic subacromial decompression for subacromial shoulder pain (CSAW): A multicentre, pragmatic, parallel group, placebo-controlled, three-group, randomised surgical trial. Lancet.

[79] Witten A., Clausen M.B., Thorborg K., Hölmich P., Barfod K.W. (2025). Bilateral ultrasonographic findings in patients with unilateral subacromial pain syndrome and intact rotator cuff tendons. J. Shoulder Elb. Surg..

[80] Brandariz R., Charbonnier C., Almeida A.C., Lädermann A., Cunningham G. (2023). The role of bone morphology of the greater tuberosity and lateral acromion on subacromial space during scaption: A three-dimensional dynamic simulation analysis. BMC Musculoskelet. Disord..

[81] Delafontaine A., Guillin R., Ropars M., Collin P. (2025). Snapping of the Subacromial Bursa: A New Cause of Shoulder Pain Demonstrated with Dynamic Ultrasound. Biomedicines.

[82] Yatish R., BK A.K., Suvarna A.A., Channappa T.S., Jayaram M., Shivakumar H.B. (2025). Effect of Ultrasound-Guided Subacromial Bursa Injections With Various Doses of Corticosteroid in Subacromial Bursitis: A Retrospective Study. Cureus.

[83] Marshall B.P., Ashinsky B.G., Ferrer X.E., Kunes J.A., Innis A.C., Luzzi A.J., Forrester L.A., Burt K.G., Lee A.J., Song L. (2024). The subacromial bursa modulates tendon healing after rotator cuff injury in rats. Sci. Transl. Med..

[84] Klatte-Schulz F., Thiele K., Scheibel M., Duda G.N., Wildemann B. (2022). Subacromial Bursa: A Neglected Tissue Is Gaining More and More Attention in Clinical and Experimental Research. Cells.

[85] Boudreau N., Gaudreault N., Roy J.-S., Bédard S., Balg F. (2019). The Addition of Glenohumeral Adductor Coactivation to a Rotator Cuff Exercise Program for Rotator Cuff Tendinopathy: A Single-Blind Randomized Controlled Trial. J. Orthop. Sports Phys. Ther..

[86] Gwilym S.E., Oag H.C.L., Tracey I., Carr A.J. (2011). Evidence that central sensitisation is present in patients with shoulder impingement syndrome and influences the outcome after surgery. J. Bone Jt. Surgery. Br. Vol..

[87] Gibson W., Wand B.M., Meads C., Catley M.J., O’COnnell N.E. (2019). Transcutaneous electrical nerve stimulation (TENS) for chronic pain-an overview of Cochrane Reviews. Cochrane Database Syst. Rev..

[88] Gleadhill C., Dooley K., Kamper S.J., Manvell N., Corrigan M., Cashin A., Birchill N., Donald B., Leyland M., Delbridge A. (2023). What does high value care for musculoskeletal conditions mean and how do you apply it in practice? A consensus statement from a research network of physiotherapists in New South Wales, Australia. BMJ Open.

[89] Başkurt Z., Başkurt F., Gelecek N., Özkan M.H. (2011). The effectiveness of scapular stabilization exercise in the patients with subacromial impingement syndrome. J. Back Musculoskelet. Rehabil..

[90] Manocchio N., Pirri C., Sorbino A., Giordani L., Vita G., Ljoka C., Foti C. (2025). Shoulder Tendinopathy Induced by Statins: A Case Report and Systematic Review. J. Pers. Med..

